# Right ventricle free wall mechanics in metabolic syndrome without type-2 diabetes: effects of a 3-month lifestyle intervention program

**DOI:** 10.1186/s12933-014-0116-9

**Published:** 2014-08-03

**Authors:** Juan Serrano-Ferrer, Guillaume Walther, Edward Crendal, Agnès Vinet, Frédéric Dutheil, Geraldine Naughton, Bruno Lesourd, Robert Chapier, Daniel Courteix, Philippe Obert

**Affiliations:** 1 Avignon University, Laboratoire de Pharm-Ecologie cardiovasculaire, LAPEC EA4278, Avignon, France; 2 Australian Catholic University, School of Exercise Science, Melbourne, Australia; 3 Blaise Pascal University, Laboratory of Metabolic Adaptations to Exercise in Physiological and Pathological Conditions (AME2P), Clermont-Ferrand, EA3533, France; 4 University Hospital of Clermont-Ferrand CHU, Clermont-Ferrand, France; 5 Omental – Thermalia Center, Châtelguyon, France

**Keywords:** Metabolic syndrome, Right ventricle, Speckle tracking, Lifestyle intervention

## Abstract

**Background:**

Growing evidence demonstrates subtle left ventricular myocardial dysfunction in patients with metabolic syndrome (MetS), with central obesity, glucose intolerance and inflammation emerging as important contributors. Whether these results can be translated to the right ventricle (RV) is not yet fully elucidated. Furthermore, although lifestyle intervention favorably impacts MetS components and inflammatory biomarkers, its effect on RV myocardial function remains unknown today.

**Methods:**

Thirty-nine MetS adults free of diabetes were enrolled in a three month lifestyle intervention program including diet and physical exercise, and compared with forty healthy controls. Blood biochemistry, echocardiography including tissue Doppler imaging (TDI), and vector velocity imaging of the RV free wall to assess global longitudinal strain (GLS) and strain rates (SR) were obtained at baseline and after the intervention.

**Results:**

Compared with controls, MetS patients presented similar right atrial and RV morphology but reduced systolic (P = 0.04) and early diastolic (P = 0.02) velocities of the tricuspid annulus. They showed attenuated RV GLS (−21.4 ± 4.5vs-25.7 ± 4.9%, P < 0.001) as well as early diastolic (P = 0.003) and systolic (P < 0.001) SR. Multiple regression analyses revealed log PAI-1 active, (P < 0.001), log adiponectin, (P = 0.01), LV mass indexed (P = 0.004) and central fat (P = 0.03) as independent predictors of RV GLS (R^2^ = 0.46, P < 0.001). Biological markers of MetS and inflammation as well as RV GLS (−21.8 ± 3.8vs-24.3 ± 3.0%, P = 0.009) and systolic (P = 0.003) and early diastolic (P = 0.01) SR, but not TDI indexes, significantly improved after diet and exercise training, and vector velocity imaging data in MetS following the lifestyle intervention no longer differed from controls.

**Conclusions:**

MetS is associated with subtle impairments in both RV free wall diastolic and systolic myocardial function which could be partly related to central-obesity induced changes in pro- and anti-inflammatory cytokines and left ventricular remodeling. The favorable impact of healthy dieting and physical activity on RV free wall mechanics indicates that cellular and sub-cellular alterations responsible for the RV myocardial abnormalities are probably not permanent and modifiable throughout adequate interventional strategies.

**Trial registration:**

American National Institutes of Health database NCT00917917.

## Background

The metabolic syndrome (MetS) is a cluster of cardio-metabolic risk factors predisposing the development of cardiovascular pathologies [1]. It is a low grade inflammatory disease [2] associated with potentially adverse effects on cardiac remodeling and function [3],[4].

Speckle tracking echocardiography is a highly sensitive technique used to detect subtle myocardial dysfunction at the preclinical stage of systolic abnormalities [5],[6]. Recent studies using this technique have reported both systolic and diastolic myocardial abnormalities in MetS patients, affecting the left ventricle predominantly in its longitudinal axis [3],[7]. Abdominal obesity, glucose intolerance and systemic inflammation biomarkers have emerged as important contributors to the depressed left ventricular longitudinal strain in MetS individuals [3],[8]. Whether right ventricular (RV) free wall mechanics are also altered in MetS remains not yet fully understood, as existing results maybe confounded by the presence of diabetes [9]-[12]. Diabetes is indeed a clinical entity, associated with altered left ventricular and RV free wall mechanics [13]-[18]. However, diabetes corresponds to an advanced stage of metabolic disorders with further potential cardiac complications due to chronic hyperglycemia, microvascular disease and autonomic neuropathy [19]. What remains to be clarified is whether MetS in the absence of diabetes leads to the development of RV myocardial dysfunction and, if present, whether these myocardial abnormalities are associated with metabolic risk and inflammatory markers. To our knowledge, only Tadic et al. [20] recently demonstrated depressed RV free wall deformations by speckle tracking imaging in non-diabetic MetS patients. The deformations were partially accounted for by hypertension, fasting glucose and abdominal obesity.

Lifestyle intervention based on dietary management and physical activity is a well-established approach to the management of various cardiometabolic diseases, including diabetes, obesity and MetS. Increasing evidence reports favorable and specific effects of lifestyle interventions on central obesity, insulin-resistance, glucose intolerance and inflammation [21]-[23]. To our knowledge, no previous studies have examined the impact of such non-pharmacological approaches on RV myocardial function in a MetS population.

Accordingly, the aims of the present study were firstly to compare RV free wall mechanics using vector velocity imaging in asymptomatic MetS adults free from type-2 diabetes, with healthy age and gender-matched controls and to evaluate in the MetS group the impact of a lifestyle intervention focused on dietary management and increased physical activity. Furthermore, we also aimed to observe relationships between RV myocardial function and metabolic risk and inflammatory markers as well as their changes consecutive to the life style intervention.

## Methods

### Study population

This study formed part of the RESOLVE trial [24]. The present experiment included 39 patients with clinically diagnosed MetS [25]. Participants with incompatible diseases such as cardiopathy, type-2 diabetes and sleep apnea syndrome were excluded. Participants with pulmonary diseases were excluded by medical history analyses, spirometry and arterial blood gas analyses. Pulmonary hypertension was excluded by verifying the absence of clinical symptoms, examination of pulmonary systolic flow profile and, in patients with detectable tricuspid regurgitation, normal pulmonary artery systolic pressure gradient [26],[27]. Coronary artery disease was excluded by verifying negative results on treadmill test and myocardial ischemia from 24-hour electrocardiographic Holter recording. MetS patients were enrolled in a 3-month lifestyle intervention program. Six participants withdrew during intervention, leaving 33 individuals for final analyses. Forty aged and gender-matched controls with no cardiovascular risk factors were recruited on a consecutive basis.

Biochemical, clinical and echocardiographic investigations were performed before and after the intervention in MetS, and at baseline only for controls. Participants provided written informed consent. The study was approved by the human ethics committee from the University Hospital of Saint-Etienne, France. The study protocol was registered with the American National Institutes of Health database N°NCT00917917.

### Biochemical, clinical and anthropometric assessment

Anthropometric variables, body composition, blood pressure and routine serum biological analyses plus N-terminal pro-B-type natriuretic peptide (NT-proBNP) were measured as previously described [3]. Insulin resistance was estimated from the homeostasis model assessment-insulin resistance (HOMA-IR) index [28]. Inflammatory markers included interleukin-6 (IL-6), high sensitivity C-reactive protein (hsCRP), adiponectin, active plasminogen activator inhibitor-1 (PAI-1 active) and tumor necrosis factor α (TNF-α).

### Lifestyle intervention

Participants were enrolled in a 3-week residential program, before returning home to continue the intervention on their own accord. Throughout the residential program, participants received daily individualized meals, planned by dietitians. Total dietary intake during the intervention was calculated to reach a 500 kcal/day caloric deficit with protein accounting for 15 to 20% and lipids 30-35%. Participants’ physical activity requirements included endurance (aquagym, cycling or walking) and resistance training (90 min, 4 to 5 days/week). Resistance training consisted of 8 free-weight exercises, performed for 3 sets of 10 repetitions. Participants were coached individually and heart rate was monitored by Polar™ S810. Participants also attended information seminars on MetS, nutrition, cooking and exercise, to support the sustainability of their new lifestyle upon returning home. Compliance to the intervention was monitored by a weekly self-reported questionnaire and phone interview conducted by dietitians.

### Echocardiography

Images were obtained by an experienced operator (GW) using a commercially available system (MyLab30, Esaote SpA, Firenze, Italy) equipped with a 3.5 MHz sector scanning electronic transducer in the left lateral decubitus position. Images at a minimum rate of 60–75 Hz were acquired in cine loops triggered to the QRS complex and saved digitally for subsequent off-line analysis with dedicated software (Mylab desk, Esaote, Italy).

### Conventional, pulsed Doppler and tissue Doppler imaging echocardiography

RV and left ventricular images were obtained following the American Society of Echocardiography guidelines [29],[26]. Left ventricular dimensions were determined from M-mode echocardiogram as previously described [3]. Left ventricular ejection fraction was obtained from the modified monoplane Simpson’s method. Left ventricular mass was calculated by the Devereux formula and indexed for height (Cornell adjustment). Pulsed-wave Doppler of mitral as well as tricuspid inflow velocities, including early (E) and atrial (A) waves, were measured. Tissue Doppler imaging (TDI) measures of myocardial systolic, early diastolic and atrial velocities were assessed at the mitral annulus level (data reported are means from lateral and septal walls) and on the lateral tricuspid annulus wall. The ratio of peak early filling velocity through the mitral valve during diastole to peak early diastolic velocity of the mitral annulus was used as an index of left ventricular filling pressure [30]. The ratio of peak early filling velocity through the tricuspid valve during diastole to peak early diastolic velocity of the lateral tricuspid annulus (E/E_tri_) and the right atrial area were used as surrogates of right atrial pressures [31],[32]. The isovolumic relaxation time for the RV was also derived from TDI measurements. The tricuspid annular plane systolic excursion (TAPSE) was measured from the difference between the displacement of RV base during systole and diastole. The fractional shortening, the right atrium area and the RV end-diastolic diameter at basal level were assessed in the apical four-chamber view. RV shortening fraction was obtained using the formula: (end-diastolic area – end systolic area)/end-diastolic area [9]. The pulmonary flow was recorded using a parasternal short-axis view at the level of the pulmonary artery allowing measurement of its acceleration time and estimation of mean pulmonary artery pressure [26].

### Vector velocity imaging

**Figure 1:**
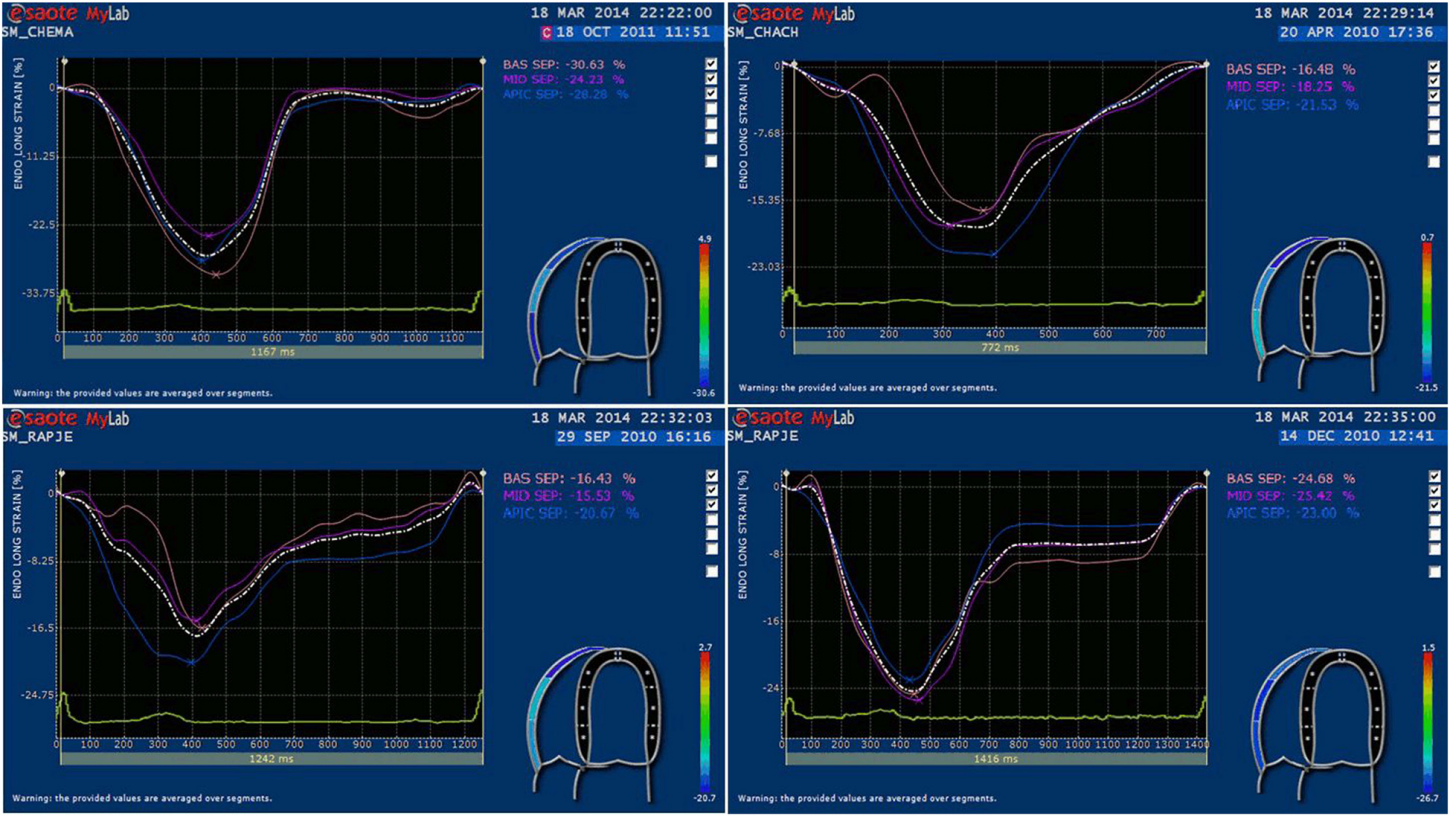
Representative curves for RV global longitudinal strain (%): one control (top left) and one MetS patient (top right), one MetS patient before (bottom left) and after (bottom right) the 3-month life style intervention program.

Velocity vector imaging is a method used to quantify myocardial wall motion through the combination of speckle tracking, tissue-blood border detection and myocardial shape [33]. It is an angle-independent measurement technique validated against sonomicrometry [33] and magnetic resonance imaging [34] which has been largely used to investigate left ventricular [3] but also RV [15],[35]-[37] mechanics in health and disease. The RV free wall longitudinal myocardial function was assessed from 2-D harmonic grey scale images in the apical 4-chamber view. The imaging sector was narrowed to optimal view and the frame rate was kept higher than 60 Hz in each case. Longitudinal strains and strain rates were measured at the basal, mid and apical segments of the free wall and averaged to give global strain (GLS) and strain rates (Figure 1). Diastolic strain rate was obtained from the peak value observed during the early filling period. To adjust all vector velocity imaging data for inter-subject differences in heart rate, a specific toolbox was used to normalize time sequence as a percentage of systolic duration calculated from the timing of pulmonary valve closure. The reproducibility of strain and strain rates (intra-observer variability: 7.8 and 8.2%, respectively) has been reported elsewhere [38].

### Statistical analysis

Statistical analyses were performed using SPSS 20.0 for Windows (SPSS Inc). Statistical significance was set a priori at P < 0.05. Variables are presented as mean ± standard derivation and skewed data were log-transformed. Appropriated unpaired and paired t-tests were used to evaluate the differences between MetS (at baseline and after the lifestyle intervention) and controls and the effect of the lifestyle intervention within the MetS patients, respectively. Mann–Whitney U-test and Wilcoxon test were used for abnormally distributed variables. For categorical variables, a χ2 test was used. Correlations using Pearson’s correlation coefficient or Spearman coefficient of rank correlation for abnormally distributed variables were used to identify associations between RV longitudinal myocardial deformation and clinical as well as biological parameters or their changes after the lifestyle intervention. Multiple stepwise linear regression analyses were performed to assess variables that were independently associated with RV free wall GLS. Biometric, metabolic and inflammatory biomarkers that correlated with RV free wall GLS with a P value <0.05 during the first univariate analysis were entered into the model.

## Results

For all parameters presented in this section, no significant differences were observed between the whole MetS group (n = 39) and the MetS subgroup (n = 33) involved in the lifestyle intervention program at baseline.

All anthropometric, biological and clinical data except height, LDL-cholesterol and NT-proBNP differed significantly between MetS and controls (Table 1). Values, excluding diastolic blood pressure, HDL-cholesterol, NT-proBNP, IL-6 and adiponectin, were reduced following the intervention program. Among biological data, glucose metabolism blood markers as well as hsCRP and TNF-α were no more significantly different from controls after the intervention program.

**Table 1:** General characteristics and clinical data

**Variables**	**Controls (n = 40)**	**MetS (n = 39)**	**MetS**	
			**Baseline (n = 33)**	**Follow-up (n = 33)**
Age (y)	58.0 ± 4.2	59.6 ± 4.6	59.4 ± 4.4	
Sex (female,%)	20 (50.0)	20 (51.2)	17 (51.5)	
MetS criteria				
Increased blood pressure (n,%)	8 (20.0)	31 (79.4)***	27 (81.8)	21 (63.6)#***
Reduced HDL (n,%)	6 (15.0)	29 (74.3)***	23 (69.7)	22 (66.6)***
Increased fasting glucose (n,%)	0 (0.0)	10 (25.6)**	7 (21.2)	2 (6.1)
Increased TG (n,%)	4 (1.0)	32 (82.0)***	27 (81.8)	22 (66.6)***
Height (cm)	169.2 ± 8.4	165.6 ± 8.2	166.1 ± 8.7	
Body weight (kg)	68.1 ± 12.4	85.9 ± 9.9***	86.0 ± 10.2	79.5 ± 9.1###***
BMI (kg.m^−2^)	23.9 ± 3.1	32.8 ± 3.5***	31.9 ± 3.5	28.8 ± 3.2###***
Waist circumference (cm)	81.9 ± 8.0	99.3 ± 7.9***	98.4 ± 7.6	91.0 ± 6.8###***
Central Fat (kg)	1.2 ± 0.5	3.0 ± 0.6***	3.0 ± 0.7	2.3 ± 0.6###***
Systolic blood pressure (mmHg)	116 ± 11	130 ± 15*	132 ± 14	120 ± 15#***
Diastolic blood pressure (mmHg)	73 ± 8	77 ± 10	76 ± 9	70 ± 9***
Heart rate (bpm)	61 ± 4	71 ± 10***	69 ± 10	65 ± 12##**
LDL-cholesterol (mmol.l^−1^)	3.6 ± 0.7	3.5 ± 0.8	3.5 ± 0.9	3.1 ± 0.8##**
HDL- cholesterol (mmol.l^−1^)	1.6 ± 0.5	1.2 ± 0.3***	1.2 ± 0.3	1.3 ± 0.4**
TG (mmol.l^−1^)	1.1 ± 0.4	2.0 ± 1.0***	1.9 ± 1.0	1.5 ± 0.6###**
Fasting glucose (mmol.l^−1^)	4.2 ± 0.5	5.0 ± 0.9***	4.9 ± .7	4.1 ± 0.6###
Fasting insulin (mIU.l^−1^)	30.2 ± 15.1	40.5 ± 13.9**	39.7 ± 14.3	35.0 ± 12.3#
HbA_1C_ (%)	5.4 ± 0.4	5.9 ± 0.4***	5.9 ± 0.3	5.7 ± 0.3###**
HOMA-IR	2.2 ± 1.3	3.6 ± 1.4***	3.5 ± 1.3	2.6 ± 1.1###
NT-proBNP (pg.ml^−1^)	22.4 ± 37.7	33.6 ± 51.3	34.3 ± 52.5	44.7 ± 79.3
IL6 (pg.ml^−1^)	1.2 ± 1.1	3.0 ± 3.5*	2.7 ± 2.8	1.9 ± 2.7
PAI-1 active (pg.ml^−1^)	7.7 ± 4.9	14.0 ± 7.1***	12.7 ± 6.4	9.5 ± 4.1##*
Adiponectin (μg.ml^−1^)	32.8 ± 22.0	17.3 ± 12.0***	17.2 ± 13.0	15.8 ± 10.6***
hsCRP (mg.l^−1^)	1.72 ± 2.77	4.03 ± 3.37***	4.12 ± 3.37	3.08 ± 3.89#
TNF-α (pg.ml^−1^)	3.5 ± 3.0	11.7 ± 8.0***	12.0 ± 8.3	5.1 ± 4.1###
Routine medication:				
Arterial hypertension (n,%)	0 (0.0)	27 (67.5)***	26 (78.8)	22 (66.7)***
ACE-I/ARBs (n,%)	0 (0.0)	20 (51.3)***	16 (48.4)	15 (45.5)***
Calcium antagonist (n,%)	0 (0.0)	3 (7.5)	3 (9.1)	3 (9.1)
Beta-blockers (n,%)	0 (0.0)	6 (15.0)*	5 (15.1)	4 (12.1)*
Diuretics (n,%)	0 (0.0)	11 (27.5)***	7 (21.2)	7 (21.2)*
Lipid lowering agents (n,%)	0 (0.0)	21 (52.5)***	16 (48.4)	15 (45.5)***

BMI: body mass index, LDL: low-density lipoprotein, HDL: high-density lipoprotein. TG: triglycerides, HbA_1C_: glycated hemoglobin, HOMA-IR: homeostatic model assessment of insulin resistance, NT-proBNP: N-terminal pro-B-type natriuretic peptide, IL6: interleukin-6, PAI-1 active: plasminogen activator inhibitor-1, hsCRP: high sensitivity C-reactive protein, TNF-α: Tumor necrosis factor α, ACE-I/ARBs: angiotensin receptor blockers and the angiotensin converting enzyme inhibitors. Significantly different from controls: *P < 0.05, **P < 0.01, ***P < 0.001. Significantly different from baseline in MetS: # P < 0.05, ## P < 0.01, ### P < 0.001.

Structure and function of the left ventricle are presented in Table 2. Increased posterior wall thickness, intra-ventricular septum thickness and left ventricular mass index were observed in MetS compared with controls. Regarding left ventricular function, mitral E/A ratio and ejection fraction were statistically lower in MetS, but the latter remained within non-pathological ranges. TDI data showed decreased early diastolic mitral annular velocity and unaltered filling pressures in MetS individuals. The lifestyle intervention program did not affect left ventricular dimensions or function in MetS, except for ejection fraction which improved after the intervention and was no longer different from controls.

**Table 2:** Left ventricle conventional echocardiography and tissue Doppler data

**Variables**	**Controls (n = 40)**	**MetS (n = 39)**	**MetS**	
			**Baseline (n = 33)**	**Follow-up (n = 33)**
LVED diameter (mm)	47.6 ± 4.9	48.9 ± 4.5	49.1 ± 4.6	49.1 ± 4.7
LVES diameter (mm)	28.9 ± 4.7	28.9 ± 4.4	28.9 ± 4.3	29.2 ± 3.7
Posterior wall thickness (mm)	9.9 ± 1.1	11.5 ± 1.6***	11.4 ± 1.4	10.9 ± 0.8***
IV septum thickness (mm)	9.9 ± 1.4	11.2 ± 1.5***	11.1 ± 1.3	10.8 ± 0.9**
LV mass indexed (g.m^-2.7^)	48.1 ± 11.9	66.1 ± 13.5***	63.7 ± 14.3	61.0 ± 10.9***
LVEF (%)	64.1 ± 5.3	61.0 ± 4.1**	61.0 ± 4.1	63.0 ± 3.1#
*Doppler data*				
Mitral E velocity (cm.s^−1^)	66.0 ± 12.2	64.4 ± 14.4	66.0 ± 11.9	64.5 ± 13.0
Mitral A velocity (cm.s^−1^)	50.4 ± 12.2	60.9 ± 15.0**	61.4 ± 15.6	57.7 ± 12.4*
Mitral E/A	1.3 ± 0.3	1.0 ± 0.2***	1.1 ± 0.2	1.1 ± 0.2*
*Tissue Doppler imaging*				
E_m_ (cm.s^−1^)	10.5 ± 2.0	9.6 ± 1.9*	9.6 ± 1.9	9.2 ± 1.0*
E/E_m_	5.9 ± 1.5	6.1 ± 1.6	6.1 ± 1.5	5.7 ± 0.9

LVED: Left ventricular end-diastolic, LVES: Left ventricular end-systolic, IV: inter-ventricular, LV: Left ventricular, EF: ejection fraction, E_m_: peak early diastolic velocity of the mitral annulus. Significantly different from controls: *P < 0.05, **P < 0.01, ***P < 0.001. Significantly different from baseline in MetS: # P < 0.05.

Structure and function of the RV are presented in Table 3. Regarding conventional data, MetS patients presented with similar right atrial and RV morphology as well as estimated mean pulmonary artery pressure, reduced TAPSE and tricuspid E/A ratio as well as greater tricuspid A compared with controls. TDI data in MetS showed decreased systolic and early diastolic tricuspid annular velocities and increased E/E_tri_ as well as isovolumic relaxation time. All these RV parameters, except RV end-diastolic dimensions, tricuspid A and E/A ratio were unaltered by the lifestyle intervention. Differences in tricuspid A and E/A ratio were no longer apparent between MetS and controls after the intervention.

**Table 3:** Right ventricle conventional echocardiography and tissue Doppler data

**Variables**	**Controls (n = 40)**	**MetS (n = 39)**	**MetS**	
			**Baseline (n = 33)**	**Follow-up (n = 33)**
RA area (cm^2^)	12.9 ± 2.0	13.0 ± 2.4	12.5 ± 1.9	12.7 ± 1.7
RVED diameter (mm)	38.8 ± 5.0	40.5 ± 5.7	39.0 ± 4.4	40.7 ± 4.5#
RV FS (%)	46 ± 5	44 ± 5	43 ± 5	44 ± 4
TAPSE (mm)	24.5 ± 2.4	22.5 ± 2.5***	22.4 ± 2.2	22.8 ± 2.1**
*Doppler data*				
Tricuspid E velocity (cm.s^−1^)	45.1 ± 7.4	46.1 ± 8.0	46.4 ± 8.0	46.6 ± 8.3
Tricuspid A velocity (cm.s^−1^)	29.2 ± 5.9	33.1 ± 7.5*	33.0 ± 8.0	29.2 ± 5.1#
Tricuspid E/A	1.6 ± 0.3	1.4 ± 0.3*	1.4 ± 0.31	1.6 ± 0.40##
Pulmonary acceleration time (ms)	157 ± 28	151 ± 30	150 ± 30	154 ± 20
Estimated mean PA pressure (mmHg)	8.4 ± 12.8	10.8 ± 13.6	9.5 ± 12.7	8.7 ± 11.2
*Tissue Doppler imaging*				
S_tri_ (cm.s^−1^)	13.5 ± 2.3	12.6 ± 2.0*	12.2 ± 2.0	11.9 ± 1.6**
E_tri_ (cm.s^−1^)	12.6 ± 2.3	11.3 ± 2.3*	11.6 ± 2.2	11.0 ± 2.3*
A_tri_ (cm.s^−1^)	14.4 ± 2.3	14.2 ± 2.4	14.1 ± 2.6	13.3 ± 3.0*
E_tri_/A_tri_	0.87 ± 0.2	0.82 ± 0.2	0.85 ± 0.2	0.88 ± 0.2
Tricuspid E/E_tri_	3.7 ± 0.8	4.2 ± 1.1*	4.1 ± 1.0	4.3 ± 1.2*
IVRT (ms)	16.3 ± 15.9	41.1 ± 26.0***	39.6 ± 23.1	33.8 ± 21.9***

RA: right atrium, RVED: right ventricular end-diastolic, RV FS: RV fractional shortening, TAPSE: tricuspid annular plane systolic excursion, PA: pulmonary artery, S_tri_: peak systolic velocity of the tricuspid annulus, E_tri:_ peak early diastolic velocity of the triscupid annulus, A_tri_: peak late diastolic velocity of the tricuspid annulus, IVRT: isovolumic relaxation time. Significantly different from controls: *P < 0.05, **P < 0.01, ***P < 0.001. Significantly different from baseline in MetS: # P < 0.05, ## P < 0.01.

Peak and time to peak values of the RV free wall longitudinal strains and strain rates are presented in Table 4. Regardless of the segments, RV free wall strains and systolic and early diastolic strain rates were lower in MetS than controls, even after control for heart rate. The intervention program not only significantly improved strains and strain rates, except for basal segment strains which failed to reach statistical significance, but reversed all data to normal control values. Timing events were similar between groups at baseline and were unaltered by the intervention.

**Table 4:** Right ventricle free wall longitudinal strains and strain-rates

**Variables**	**Controls (n = 40)**	**MetS (n = 39)**	**MetS**	
			**Baseline (n = 33)**	**Follow-up (n = 33)**
RV strain (%)				
RV GLS	−25.7 ± 4.9	−21.4 ± 4.5***	−21.8 ± 3.8	−24.3 ± 4.0##
Basal segment	−27.1 ± 7.1	−23.4 ± 5.1*	−23.7 ± 4.7	−24.7 ± 7.0
Mid segment	−25.4 ± 5.4	−20.9 ± 5.0***	−21.1 ± 4.7	−23.6 ± 4.2#
Apical segment	−24.7 ± 6.1	−19.7 ± 6.0***	−20.1 ± 5.8	−23.7 ± 5.4##
RV GLS TTP (ms)	94 ± 6	92 ± 10	93 ± 10	93 ± 8
RV global systolic SR (.s^−1^)	−1.8 ± 0.4	−1.2 ± 0.4***	−1.2 ± 0.4	−1.5 ± 0.4##
RV global systolic SR TTP (ms)	51 ± 6	50 ± 9	48 ± 9	50. ± 10
RV global diastolic SR (.s^−1^)	1.7 ± 0.5	1.3 ± 0.3**	1.3 ± 0.3	1.6 ± 0.4##
RV global diastolic SR TTP (ms)	123 ± 10	121 ± 16	122 ± 16	118 ± 14

GLS: global longitudinal strain calculated from the mean of the basal, mid and apical segments of the right ventricle free wall, RV: right ventricular, SR: strain rate, TTP: time to peak. Significantly different from controls: *P < 0.05, **P < 0.01, ***P < 0.001. Significantly different from baseline in MetS: # P < 0.05, ## P < 0.01.

Significant correlations were obtained between RV GLS and markers of central obesity, glucose intolerance and inflammation as well as left ventricular hypertrophy (Table 5). Log PAI-1 active (β = 0.51, P < 0.001), Log adiponectin, (β = −0.27, P = 0.01), left ventricular mass indexed (β = 0.34, P = 0.004) and central fat (β = 0.29, P = 0.03) emerged as independent predictors of RV GLS, explaining 46% of its variance (P < 0.001). However, no relationships were observed between delta changes of RV GLS with other MetS components or inflammatory markers following the lifestyle intervention (data not shown).

**Table 5:** Relationship between RV free wall global strain and clinical, biological and echocardiographic data

**Variables**	***Univariate analysis***	
	***r***	***P***
Age (Years)	0.15	ns
Systolic blood pressure (mmHg)	0.12	ns
Diastolic blood pressure (mmHg)	0.14	ns
Central fat	0.32	**
Waist circumference	0.45	***
BMI	0.39	***
HDL	−0.39	***
LDL	−0.05	ns
Triglycerides	0.12	ns
HbA_1C_	0.36	**
Fasting glucose	0.35	**
HOMA-IR	0.22	*
TNF-α	0.31	*
PAI-1 active	0.52	***
IL-6	0.30	**
Adiponectin	−0.53	***
Left ventricular mass indexed	0.43	***
E_tri_	−0.37	**
S_tri_	−0.26	*
E/E_tri_	0.39	**

BMI: Body mass index, LDL: low-density lipoprotein, HDL: high-density lipoprotein, HbA_1C_: glycated hemoglobin, HOMA-IR: homeostatic model assessment of insulin resistance, TNF-α: Tumor necrosis factor α, PAI-1 active: plasminogen activator inhibitor-1, IL6: interleukin-6, E_tri:_ peak early diastolic velocity of the tricuspid annulus, S_tri_: peak systolic velocity of the tricuspid annulus. Significant differences categorized as: *P < 0.05, **P < 0.01, ***P < 0.001. ns: non-significant.

## Discussion

We provide evidence for the first time of non-diabetic MetS patients exhibiting subtle RV free wall systolic and diastolic dysfunction that was significantly improved following a 3-month lifestyle intervention based on healthy dieting and increased physical activity.

Currently, a large body of evidence associates MetS with adverse effects on left ventricular myocardial function assessed using tissue deformation imaging tools [3]. However, far less information is available on RV myocardial function and whether the latter is sparse in the settings of MetS remains largely unresolved. Most of the available studies refer to assessment of RV free wall velocities by TDI and report inconclusive results, especially for longitudinal systolic velocities [9]-[11],[20]. Our data of reduced systolic and early diastolic longitudinal velocities agree with some of these works [10],[11] but not all [9],[20]. A major limitation of TDI over speckle tracking imaging is however, its lack of sensitivity (dependency on loading conditions, tethering effect, translational cardiac movement) in detecting subtle myocardial changes [3],[37],[39]. This is of particular concern when evaluating systolic abnormalities but also the impact of interventional strategies (see paragraph below). Additionally, due to insonation angle-dependency, TDI data are usually restricted generally to basal and sometimes mid segments of the left or right ventricle. Subsequently, only a partial understanding is permitted of the effect of MetS on regional myocardial performance. Using sensitive echocardiographic tolls such as speckle tracking imaging surpasses most of the TDI aforementioned limitations [39]. Speckle tracking imaging is feasible and applicable to the RV and has been shown to provide extensive information about RV myocardial function in various cardiometabolic disease [15],[20],[37],[40]. In the present study, we used vector velocity imaging, a technique that has been validated for accurate assessment of myocardial deformation [33],[41] to fully explore the entire RV free wall mechanics. We demonstrated subtle alterations of both systolic and diastolic linear deformations encompassing all segments of the RV free wall in MetS patients free of type 2 diabetes. These results agree with those recently published by Tadic et al. [20] in MetS individuals also free of diabetes.

Abdominal obesity, a key component of MetS, is consistently associated with major increases in pro-inflammatory adipocytokines, such as TNF-α, IL-6, hsCRP or PAI-1 active, as well as reduced protective cytokines, such as adiponectin, agreeing with our data [42]-[44]. Growing evidence suggests a pivotal role of visceral adipose tissue to the left ventricular myocardial dysfunction observed in various metabolic diseases, postulated to occur via a low-grade state of inflammation [45]-[47]. Whether this is also true for RV myocardial dysfunction in MetS remains largely unknown. To our knowledge, only Tadic et al. [20] documented from univariate and multivariate regression analysis independent associations between global RV free wall deformations and some MetS criteria including waist circumference, fasting glucose and systolic blood pressure in MetS individuals free of diabetes. The results of the present study confirm and extend these results by demonstrating significant relationships between RV GLS and abdominal obesity as well as inflammatory biomarkers. From multivariate analysis, central fat, PAI-1 active as well as adiponectin, appeared as significant contributors to RV free wall dysfunction. Central adipose tissue-induced inflammation might have precipitated the RV free wall myocardial abnormalities reported here via enhanced oxidative stress, adversely affecting coronary endothelial function as well as impairing cardiomyocyte calcium handling and increasing fibrosis [47]-[49]. Of note, adiponectin exerts cardiovascular protective effects via its ability to limit apoptosis, oxidative stress and inflammation in cardiomyocytes and endothelial cells [42]. Nonetheless, the depressed RV GLS may also be explained by ventricular interdependence, and specifically, left ventricular hypertrophic remodeling, through direct mechanical interactions between the two chambers. As previously demonstrated, left ventricular hypertrophy and dilation (a remodeling classically observed in MetS [3] and in the present population), result in RV compression leading in turn to impaired function [50]. Supporting this assumption, not only indexed left ventricular mass correlated with RV GLS but also appeared as one of its main contributors from stepwise multiple regression analysis.

To the best of our knowledge, no studies have examined the effect on RV myocardial function of non-pharmacological interventional strategies in MetS populations. The other major novel finding from the present study was that a 3-month lifestyle intervention comprising nutrition and exercise training was able to fully restore RV GLS to normal values of age-matched healthy controls. Of note, improvements in RV free wall function were evidenced only using sensitive tools such as vector velocity imaging, as TDI indices were not changed. RV myocardial function enhancements have also been reported with obesity following interventions involving low calorie diet [51]. Agreeing with previous data [22], the lifestyle intervention favorably impacted on abdominal obesity, glucose intolerance and most biomarkers of inflammation. Despite significant relationships between RV GLS and most of MetS components at univariate analysis, no correlations were noticed between relative change data after the intervention. This could be due in part to the low magnitude of changes observed and a relatively small sample size, although patients acted as their own controls. Accumulation of ectopic fat to the heart is emerging as the key component of myocardial dysfunction in metabolic diseases [12],[47],[52]. Increased epicardial fat, as established in MetS [53] is a local source of pro- and anti-inflammatory cytokines whereas visceral abdominal fat is mainly responsible for the increased systemic inflammation [42],[44]. Although not measured in the present study, observed improvements in the present study could be attributed to diet and exercise-induced reduction in cardiac adiposity, in turn lowering local inflammation and oxidative stress. Of note, a low calorie diet program in 20 severely obese patients [54] decreased epicardial fat more than in other sites of adipose tissue and improvement in LV diastolic function was more strongly related with epicardial fat changes than with other adiposity indices.

Despite its key role in left ventricular filling, RV function has been insufficiently investigated in cardiometabolic diseases. With prevalence reaching alarming proportions worldwide, MetS is now considered to be the driving force for a cardiovascular disease epidemic. In this context, the present study underlines the necessity for a close clinical RV monitoring in MetS patients even when type 2 diabetes is not associated. Moreover, this study underlines the importance of central obesity and its associated inflammation as independent factors explaining RV mechanical abnormalities, highlighting the need for treatment of central fat and inflammation to decrease or prevent the deleterious impact of MetS on RV function. Finally, the present work emphasizes the importance of lifestyle changes since the RV dysfunction can be corrected even only three months after an exercise and nutrition intervention.

### Study limitations

A first limitation of the present study is its relatively small sample size. Additionally, most of our MetS patients presented with arterial hypertension and half were on ACE-I/ARBs, which considering previous studies [18],[20],[55] could have confounded our results on RV free wall function. However, blood pressure did not correlate with RV GLS, nor did it emerge as an independent contributor in multivariate analysis. Additionally, there were no differences in RV free wall mechanics indices between subgroups of MetS treated or not for hypertension. Interestingly, Gökdeniz et al. [12] did not shown in MetS patients any contributing effect of coexisting hypertension to RV free wall longitudinal strain by speckle tracking imaging. In contrast, systolic blood pressure emerged as an independent contributor to the altered global RV free wall strains reported by Tadic et al. [20] in non-diabetic MetS patients. Further studies will therefore be needed to clarify the independent effect of mild to moderate hypertension, as encountered in MetS, on RV free wall mechanics. Manipulation of the renin-angiotensin-aldosterone system via ACE-I/ARBs has been shown to affect RV remodeling and possibly RV function [55],[56]. It is however, unlikely that ACE-I/ARBs influenced our results since no differences in RV GLS were observed between the subgroups of MetS patients taking or not ACE-I/ARBs medication. Right atrial pressure is an important component of RV function [32]. In our study, right atrial pressures were not invasively measured through right heart catheterization due to ethical considerations. Specifically, pressures were estimated from E/E_tri_ and right atrial area. Although significant, the differences between the 2 groups in E/E_tri_ were low (3.7 ± 0.8 vs 4.2 ± 1.1, P = 0.02) and most importantly only one MetS patient out of 39 presented with a ratio greater than 6; a cut-off value proposed by Nagueh et al. [31] that can be considered to be a marker of RA pressures greater than 10 mmHg. Furthermore, no right atrial remodeling was observed in our MetS patients and E/E_tri_ failed to correlate with right atrial area (r = 0.06 P = 0.62). Collectively, these results favor the absence of elevated right atrial pressures in our MetS patients. Subsequently it is unlikely that increased right atrial pressures could have accounted for their reduced RV GLS. Of note, stepwise multiple regression results indicated that E/E_tri_ was not a significant contributor to the RV GLS. Additionally, no changes in E/E_tri_ ratio but also right atrial area were noticed following the lifestyle intervention program while at the same time, the latter fully restored RV GLS to normal values, demonstrating that variables were not independently associated. As previously noted, cardiac adiposity was not measured and whether it is involved in the RV myocardial abnormalities reported here and whether improvement in RV function after lifestyle intervention are linked to favorable impacts on myocardial steatosis and/or epicardial fat remains to be determined. Of note, Gökdeniz et al. [12] recently demonstrated in MetS patients using speckle tracking imaging, that epicardial fat was independently associated with RV free wall global longitudinal strain.

In conclusion, RV myocardial systolic and diastolic abnormalities in MetS patients free of type-2 diabetes were partially accounted for by central adiposity-induced changes in pro- and anti-inflammatory cytokines as well as ventricular interdependence, through direct mechanical interactions between the RV and left ventricular chambers. A lifestyle intervention based on healthy dieting and physical activity was associated with fully restored RV free wall mechanics to healthy control level; indicating probably that cellular and sub-cellular alterations were not permanent but still modifiable throughout adequate interventional strategies. Special attention should be paid to this specific population in clinics, as earlier identification of asymptomatic patients at high risk of evolution to RV failure is of primary importance because it may facilitate timely and more effective intervention.

## Abbreviations

E/Etri: Ratio of peak early filling velocity through the tricuspid valve during diastole to peak early diastolic velocity of the lateral tricuspid annulus

HbA1C: Glycated hemoglobin

HDL: High-density lipoprotein

HOMA-IR: Homeostatic model assessment of insulin resistance

hsCRP: High sensitivity C-reactive protein

IL6: Interleukin-6

LDL: Low-density lipoprotein

MetS: Metabolic syndrome

NT-proBNP: N-terminal pro-B-type natriuretic peptide

PAI-1 active: Active plasminogen activator inhibitor-1

RV: Right ventricle

RV GLS: Global longitudinal strain of the right ventricular free wall

TAPSE: Tricuspid annular plane systolic excursion

TDI: Tissue Doppler imaging

TG: Triglycerides

TNF-α: Tumor necrosis factor α

## Competing interests

The authors declare that they have no competing interests.

## Authors’ contributions

PO is the guarantor of the entire manuscript. JS-F contributed to analysis and interpretation of data, drafting of the manuscript, and critical revision of the manuscript for important intellectual content, and takes responsibility for the integrity of the work as a whole. GW contributed to acquisition, analysis and interpretation of data, drafting of the manuscript, and critical revision of the manuscript for important intellectual content, and takes responsibility for the integrity of the work as a whole. EC contributed to analysis and interpretation of data, and critical revision of the manuscript for important intellectual content. AV contributed to analysis and interpretation of data, and critical revision of the manuscript for important intellectual content. FD contributed to study concept and design, acquisition of data, drafting of the manuscript and critical revision of the manuscript for important intellectual content. GN contributed to interpretation of data, drafting of the manuscript, and critical revision of the manuscript for important intellectual content. BL contributed to study concept and design, analysis of data and critical revision of the manuscript for important intellectual content. RC contributed to study concept and design, interpretation of data and critical revision of the manuscript for important intellectual content. DC contributed to study concept and design, to analysis and interpretation of data, and critical revision of the manuscript for important intellectual content. PO contributed to study concept and design, to acquisition, analysis and interpretation of data, drafting of the manuscript, and critical revision of the manuscript for important intellectual content. All authors read and approved the final manuscript.
